# Evaluating the effectiveness of the violence against persons prohibition act in reducing female genital mutilation/cutting in Nigeria: a multi-year policy impact analysis

**DOI:** 10.1186/s13104-026-07700-1

**Published:** 2026-02-08

**Authors:** Emmanuella C. Nzeribe, Iyobosa E. Amadin, Olugbemisola W. Samuel, Stephen O. Asaolu, Evelyn E. Orya, Laila Umar, Deborah C. Okoli, Hilary I. Okagbue

**Affiliations:** 1 Sydani Institute of Research and Innovation, Sydani Group, Abuja, Nigeria; 2 Sydani Group, Abuja, Nigeria; 3 National Primary Healthcare Development Agency, Garki, Abuja, Nigeria; 4 Department of Mathematical Sciences, Mississippi State University, Starkville, USA

**Keywords:** Female genital mutilation, Genital cutting, Legislation, Sexual violence, Nigeria, UNICEF MICS

## Abstract

**Background:**

Female genital mutilation/cutting (FGM/C) is a harmful practice that is performed in various parts of the world, especially in Africa, the Middle East, and Asia. Although the Federal Government of Nigeria signed the Violence Against Persons Prohibition (VAPP) Act into law in 2015, little is known about the extent to which the law has contributed to reducing the practice of FGM/C in Nigeria. Therefore, this paper seeks to compare the prevalence of FGM/C before and after the enactment of legislation prohibiting it in Nigeria using data from the Multiple Indicator Cluster Surveys (MICS) of 2007, 2011, 2016/2017, and 2021 (individual recode file), which collected data from women aged 15–49 years across the 36 states of Nigeria, including the Federal Capital Territory (FCT).

**Results:**

The study findings showed that awareness of FGM/C was high across the four (4) rounds of MICS survey datasets, while the prevalence of mothers and their daughters being circumcised has declined. The prevalence of FGM/C practice is lowest in the northeastern region and has been declining steadily in the southwestern region. FGM/C practices are still relatively high in the southeast, southwest, north-central, and northwest, respectively. The findings indicate statistically significant differences in awareness and prevalence of FGM/C before and after the enactment of the VAPP Act, with heterogeneous patterns across geopolitical regions. While these trends are consistent with a potential policy effect, the results should be interpreted as associative rather than causal.

## Background

It is estimated that over 200 million girls and women have been subjected to female genital mutilation/cutting (FGM/C) at some point in their lives, and approximately three million girls currently face the risk of undergoing the procedure annually [1]. The practice is most prevalent in Africa, where it continues to be performed in more than 30 countries [2]. FGM/C has deep societal roots and is often viewed as a necessary step in preparing young girls for adulthood and, subsequently, marriage [3]. According to global estimates [4], 125 million women and girls have experienced FGM/C, accounting for approximately 10% of the global female population. Several prevalences across Africa have been reported [5]– [6].

The practice of FGM in Nigeria is widespread and varies from one geopolitical region (GPR) to another. The highest prevalence of FGM is reported in the Southeast and Southwest GPRs of the country [7]. Although the commonest types practiced in Nigeria are types I and II [7], the other types of FGM (types III and IV) are also carried out, particularly in the northern parts of Nigeria [7]. Studies from empirical literature and findings from the Demographic Health Surveys (DHS) and the UNICEF Multiple Indicator Cluster Survey (MICS) data on FGM/C have shown varying prevalences of the practice in Nigeria [7, 8]. Similarly, findings from other nationally representative surveys have shown little change in the attitude towards continuation of FGM/C among Nigerian women aged 15 to 49 [7, 9].

FGM/C violates several human rights outlined under the Universal Declaration of Human Rights, the Convention on the Elimination of All Forms of Discrimination against Women, and the Convention on the Rights of the Child [10]. Legislation is a powerful tool in the fight against FGM/C by providing a formal mechanism to prohibit the practice, protect victims, and hold perpetrators accountable. However, its success depends on effective enforcement, public awareness, and integration with broader social and cultural initiatives.

The Federal Government of Nigeria recognized FGM/C as a discriminatory act that requires legislation, which led to the national female genital mutilation (FGM) policy. The first federal law enacted to prohibit FGM/C across the country is the Violence Against Persons (Prohibition) Act (VAPP), which came into force on 25 May 2015 [11]– [12]. VAPP Act prohibits female circumcision or genital mutilation and any person who performs or engages another to carry out such circumcision [12].

This Act ought to be adopted by all the states in Nigeria; however, as of April 2024, only 3 states, Kano, Rivers, and Taraba, are yet to pass the VAPP Act into law [13]. Extant studies have posited that a combination of legislation and political will could be effective in changing attitudes towards FGM and its prevalence [14]. Also, previous studies focus on prevalence patterns or sociocultural determinants without explicitly examining temporal changes before and after the enactment of the VAPP Act using nationally representative data. In the absence of any published work that tied the VAPP Act and the prevalence of FGM/C in Nigeria, the study seeks to assess the impact of the VAPP Act by looking at the trend of FGM/C before and after the enactment of the legislation criminalizing FGM/C practices in Nigeria. To address the study objective, the following hypotheses were tested:

- H1: Awareness of FGM/C among women aged 15–49 years differs between the pre-VAPP Act and post-VAPP Act periods.
- H2: The prevalence of FGM/C among women aged 15–49 years differs between the pre-VAPP Act and post-VAPP Act periods.
- H3: The prevalence of daughters being circumcised differs between the pre-VAPP Act and post-VAPP Act periods.
- H4: The prevalence of FGM/C differs between states that have domesticated the VAPP Act and those that have not.
- H5: In states yet to domesticate the VAPP Act, there is no significant difference in FGM/C prevalence before and after national passage of the Act.

## Methodology

### Data source

This study adopted the MICS of 2007, 2011, 2016/2017 and 2021. The MICS data are cross-sectional surveys that provides indicators on children and women at the national, rural/urban, states and the six geopolitical zone levels in Nigeria.

. The MICS data comprise household surveys implemented by countries under the program developed by the United Nations Children’s Fund (UNICEF).

### Study setting

The study was conducted in both urban and rural areas of Nigeria. The data was collected from all 36 states in Nigeria and the Federal Capital Territory.

### Study design

The study used a retrospective cross-sectional design based on secondary data retrieved from Nigeria’s Multiple Indicator Cluster Surveys and other household surveys conducted by countries participating in a UNICEF-supported program.

### Target population and sample size

The target population in this study was adolescent girls and women of reproductive age. The sample size of the adolescent girls and women from the selected 4 rounds in this study dependent on the variables as presented in Table 1.

### Data collection tool

UNICEF designed the questionnaires to collect information on various indicators, but this study extracted only selected data on specific aspects of FGM/C for women aged 15–49 years. The data used in this research were obtained from publicly available UNICEF datasets.

### Variables

#### Outcome variables

The variables obtained were similar across the years, and they include ‘ever heard of circumcision’ (EHC), ‘ever circumcised’ (EC), and ‘daughter circumcised’ (DC). The binary responses of whether the respondent was circumcised (yes/no) were used as the dependent variables. However, responses in the third category were classified as missing data.

#### Independent variable

The geopolitical region (GPR) in the UNICEF Nigeria MICS was the only independent variable extracted from the four datasets. The six GPRs were North Central (NC), North East (NE), North West (NW), South East (SE), South South (SS), and South West (SW). This choice reflected the study’s aim to categorize outcome variables by GPR and to track and compare prevalence across the zones.

### Data analysis

These variables were analyzed using SPSS software version 27. All the data were weighted to account for disproportionate sampling and non-response, taking into consideration the multi-stage sampling design of the survey. Missing values were excluded from the analysis, therefore no imputation was performed. The data was rescaled, and the missing data are presented in Table 1.

**Table 1:** The various sample sizes and their corresponding missing data

Variable		2007		2011		2016/2017		2021
	Valid	Missing	Valid	Missing	Valid	Missing	Valid	Missing
EHC	24,563	2530	20,543	0	22,274	5	24,826	10
EC	12,649	14,444	20,529	14	21,740	539	23,476	1360
DC	7041	20,052	15,323	5220	17,078	5201	17,772	7064
GPR	27,093	0	20,543	0	22,279	0	24,836	0

Non-parametric statistical tests were employed because the outcome variables were binary and the distributions violated assumptions of normality. The Kruskal–Wallis and Jonckheere–Terpstra tests were used to assess differences and temporal trends across survey years, while the Mann–Whitney U test compared FGM/C prevalence between states that had domesticated the VAPP Act and those that had not.

## Results

### Prevalence of FGM/C in Nigeria

The result, as presented in Table 2, indicated that there was a higher ‘Yes’ answer to awareness about FGM/C, and the percentage of women who have heard about FGM/C increased from 2007 to 2011, with a slight decrease of 1.4% and 1.5% in 2016/2017 and 2021 respectively. Moreover, the number of females that were circumcised between 2007 and 2011 increased from 42.2% to 46.5%, which later declined in 2016/2017 and further declined in 2021. More daughters of the respondents were circumcised in 2016/2017 than any other year, with the lowest percentage being in 2021. The percentage of daughters circumcised is lowest in 2021 and highest in 2016/2017. Overall awareness of FGM/C remained high across survey years, while the prevalence among women and their daughters showed a declining trend.

**Table 2:** Prevalence of FGM/C in Nigeria from 2007 to 2021

Variables	2007 (%)	2011 (%)	2016/7 (%)	2021 (%)
**EHC**				
Yes	12,415 (50.5)	20,032 (97.5)	21,329 (95.8)	23,417 (94.3)
No	12,148 (49.5)	511 (2.5)	945 (4.2)	1409 (5.7)
**EC**				
Yes	5335 (42.2)	9556 (46.5)	9274 (42.7)	7745 (33.0)
No	7314 (57.8(	10,973 (53.5)	12,466 (57.3)	15,731 (67.0)
**DC**				
Yes	1337 (19.0)	2585 (16.9)	4075 (23.9)	2197 (12.4)
No	5704 (81.0)	12,738 (83.1)	13,003 (76.1)	15,575 (87.6)

### Prevalence of FGM/C across the GPRs

Prevalence data disaggregated by geopolitical region (GPR) show marked regional variation **(**Table 3**)**. The South-West (SW) records the highest awareness of FGM/C, exceeding 90% across all survey periods, while awareness slightly declined in the North-West (NW) but increased steadily in the South-East (SE) from 89.3% in 2007 to 99.0% in 2021. FGM/C prevalence is lowest in the North-East (NE), declining from 6.2% to 3.1%, and also decreased in the SW from 61.7% to 51.5%. Southern regions report higher prevalence overall, though gradual increases are observed in the North-Central and NW. Circumcision of daughters is declining nationally, nearly absent in the NE and most prevalent in the NW.

**Table 3:** Prevalence of FGM/C in Nigeria from 2007 to 2021across the GPRs

EHC	2007 (%)	2011 (%)	2016/7 (%)	2021 (%)
NC	2030 (44.4)	3443 (97.4)	3397 (94.9)	4158 (96.8)
NE	1163 (23.3)	2209 (97.7)	1686 (93.2)	3844 (93.0)
NW	1115 (19.2)	2710 (96.7)	5191 (92.2)	3519 (88.5)
SE	2541 (89.3)	3685 (98.9)	2990 (99.5)	4064 (99.0)
SS	3091 (85.6)	4028 (95.0)	4088 (96.7)	3862 (90.1)
SW	2475 (90.2)	3957 (99.4)	3977 (98.9)	3970 (98.4)
**EC**				
NC	629 (30.2)	1318 (37.3)	911 (25.6)	1236 (29.6)
NE	74 (6.2)	98 (4.3)	59 (3.3)	127 (3.1)
NW	110 (9.5)	953 (34.1)	2681 (50.7)	918 (26.4)
SE	1533 (59.2)	2472 (66.4)	1549 (52.6)	1979 (50.6)
SS	1443 (46.1)	2147 (50.7)	1799 (42.7)	1581 (37.7)
SW	1546 (61.7)	2568 (64.5)	2275 (57.9)	1904 (51.5)
**DC**				
NC	184 (14.6)	266 (10.0)	392 (14.1)	374 (12.2)
NE	14 (2.0)	60 (3.3)	36 (2.6)	26 (0.8)
NW	45 (5.7)	754 (35.0)	2525 (56.1)	1054 (36.3)
SE	272 (23.4)	365 (13.4)	265 (11.9)	166 (5.7)
SS	286 (16.3)	331 (10.8)	206 (6.6)	136 (4.6)
SW	536 (39.1)	809 (27.4)	651 (21.4)	441 (15.3)

### Test of equality of medians

Kruskal-Wallis and Jonckheere-Terpstra tests showed that the median EHC, EC, and DC differed significantly over the four periods (Table 4). The respective hypotheses’ alternatives are accepted. Hence, the following interpretations are deduced from Table 4.

**Table 4:** Test results for the hypotheses

Variable	Kruskal-Wallis	Jonckheere-Terpstra
EHC	H = 27116.814, *p* < 0.001	JT = 1,231,462,041, *p* < 0.001
EC	H = 914.957, *p* < 0.001	JT = 1,208,429,789, *p* < 0.001
DC	H = 803.023, *p* < 0.001	JT = 612,213,906, *p* < 0.001

1. The awareness level of FGM/C is different before and after the VAPP Act
2. The prevalence of FGM/C is different before and after the VAPP Act, and
3. The prevalence of daughters being circumcised is different before and after the VAPP Act.

### FGM prevalence differences between states that passed the policy and those that did not

According to the VAPP tracker, Taraba, Rivers, and Kano states have yet to adopt, ratify, and pass the VAPP Act into law. The EC was disaggregated based on the states that have passed the law versus those that are yet to pass the law, and two hypotheses were formulated.

A Mann-Whitney test was used. The null of the fourth hypothesis was rejected (W = 375963318, p-value < 0.00001), while the null of the fifth hypothesis was accepted (U = 925904, p-value = 0.683510).

## Discussion

The observed differences in FGM/C awareness and prevalence before and after the enactment of the VAPP Act are consistent with potential policy influence, although causality cannot be established due to the study design.

### Increase in awareness of FGM/C in Nigeria

There was an increase in the awareness level of FGM/C. The southern part of the country has a higher awareness of FGM/C compared to the northern part. The research corroborated a study that reported a 98.7% awareness level among 367 women in the South-east region of the country [15]. A decline in the awareness level in North-west Nigeria was also observed. The increasing awareness of FGM/C in Nigeria is a positive development and points to the effectiveness of several interventions in creating awareness of these harmful practices. Simply put, people are increasingly becoming aware of the dangers and effects of this harmful practice [16]. However, the disparity in awareness levels between the Southern and Northern regions [17], particularly the decline in the North-west, raises significant concerns that must be addressed by policymakers. The southern part of Nigeria, which has historically shown higher literacy rates and greater access to healthcare and information as posited by several authors [16], reflects a higher level of awareness of different health outcomes such as FGM/C. The disparities are not limited to indicators such as healthcare utilization but also extend to sexual and reproductive health [18], and nutritional status [19]. This suggests that efforts to educate the public about the dangers of FGM/C have been more successful in the South-west, South-east [15], and South-south GPRs, likely due to better infrastructure, stronger community engagement, and more robust implementation of anti-FGM/C policies.

### Decrease in the prevalence of FGM/C in Nigeria

The research found a decreasing prevalence of FGM/C in Nigeria, for which the VAPP Act could be a contributory factor. Further research is needed to determine whether certain variables in the MICS data are intervening or moderating factors in the observed decline. Moreover, this declining trend has been previously reported in Nigeria by [7], in other regions across Africa and Western Asia [20], and globally [5]. The SW region in Nigeria, traditionally a high-prevalence area, has seen a steady decline from 61.7% in 2007 to 51.5% in 2021. Similarly, the NE region has experienced a marked reduction from 6.2% in 2007 to 3.1% in 2021. These trends suggest that cultural attitudes towards FGM/C are changing, and more families are choosing to abandon the practice [21]. The Northeast region has consistently shown the lowest prevalence of FGM/C, indicating that interventions in this area have been particularly effective or that other factors, yet to be reported in the literature, may be at play. While the Southern region initially had higher rates, the steady decline demonstrates that efforts by policymakers to address this issue are gaining traction. Additionally, the observed narrowing gap between the Southern and Northern regions suggests that the prevalence of FGM/C is becoming more evenly distributed across the country.

### Decrease in the prevalence of FGM/C for daughters of mothers

Generally, there is a decline in the prevalence of circumcising young girls, corroborating an earlier study [20]. This research has shown a “generational shift” in the prevalence of FGM/C in Nigeria in two ways. First, all the other GPRs except the northwest have seen a steady decline in the prevalence of FGM/C among daughters of respondents. However, there is an increase in the prevalence in the northwest region, which could be due to cultural and religious influences, limited access to healthcare, education, and social services, and social pressures and stigma. A study has reported that girls in the North-west have high odds of being cut [8].

The decrease in FGM/C prevalence among daughters of respondents in five out of the six GPR is a positive indicator of changing attitudes and practices, however, the decline is slower than required to produce a substantial impact [22]. Hence, studies have been reporting the prevalence of FGM/C to be higher in older women than younger ones for southeast [15] and northcentral [23]. This suggests that efforts to raise awareness and promote alternative cultural norms are having a tangible impact, as young girls are reporting higher awareness of FGM/C compared with older women [24]. Other factors are increased access to education, particularly for girls, rapid urbanization, community-based initiatives, and other forms of intervention [25].

### The impact of the VAPP act

According to the VAPP tracker (as of August 21, 2024), 34 out of 36 states and the FCT have signed the VAPP act into law [13]. The research indicating differences in FGM/C awareness and prevalence among mothers and their daughters before and after the implementation of the VAPP Act suggests that the legislation could have played a role, although the observed relationship vary across the six GPRs.

The adoption of the VAPP Act in most Nigerian states has likely contributed to a sustained high level of awareness of FGM/C and its harmful and deleterious effects, especially in sexual and reproductive health (SRH) [26] and mental health [27]. The research showing differences in awareness levels before and after the VAPP Act suggests that the legislation might have contributed to bringing the issue of FGM/C to the forefront of public discourse. While FGM/C awareness has generally increased in the country, the level of awareness varied significantly between different regions and generations, as shown in this research, particularly in regions where FGM/C was more deeply entrenched, such as parts of the South where most of the states had earlier signed the VAPP Act.

The research indicates that the prevalence of FGM/C has decreased among daughters compared to their mothers, suggesting a generational shift probably influenced by the VAPP Act. The VAPP Act’s prohibition of FGM/C, along with penalties for those who perform or facilitate the practice, has likely deterred many families from continuing the practice with the younger generation.

Finally, this research has shown that the states that passed the VAPP Act indicate strong evidence against the null hypothesis, meaning there is a significant effect, likely reflecting a reduction in FGM/C prevalence. On the other hand, the states without the law indicate no significant difference, meaning the absence of the VAPP Act has not had a discernible effect on FGM/C prevalence. However, this result must be interpreted with caution for two reasons. One, other drivers apart from the VAPP Act could be key influencing factors, and lastly, sufficient evidence for strong justification is lacking due to limitations in study design.

## Limitation

This study has several limitations. First, the repeated cross-sectional design limits causal inference and does not allow tracking of individual-level behavioral change over time. Second, FGM/C status was self-reported and may be subject to recall or social desirability bias, particularly in the post-VAPP period when legal sanctions may discourage disclosure. Third, the analysis did not adjust for potential confounders such as education, wealth, religion, or urban–rural residence, which may independently influence FGM/C practices.

## Conclusion

In conclusion, this study provides a national overview of trends in FGM/C prevalence and awareness in Nigeria, showing a steady decline in prevalence alongside increasing awareness over time. The stronger declines among younger generations suggest a generational shift, with many mothers choosing not to continue the practice, reflecting changing social norms and attitudes. However, persistently higher prevalence and declining awareness in the North-West reveal important regional disparities that require context-specific responses. Using four rounds of UNICEF MICS data, this study offers one of the first national, multi-wave assessments in the context of the VAPP Act, providing evidence to inform policy, programming, and resource allocation, while recognising that observed trends are associative rather than causal.

## Data Availability

The data that support the findings of this study are publicly available at UNICEF Multiple Indicator Cluster Survey (MICS) website once registered. The Accession numbers are as follows: NGA_2007_MICS_v01_M NGA_2011_MICS_v01_M NGA_2016_MICS_v01_M NGA_2021_MICS_v01_M.

## Abbreviations

DHS: Demographic Health Survey
FCT: Federal Capital Territory
FGM/C: Female Genital Mutilation/Cutting
GBV: Gender-Based Violence
GPR: Geopolitical Region
LE: Life Expectancy
MICS: Multiple Indicator Cluster Survey
SDG: Sustainable Development Goals
SRH: Sexual and Reproductive Health
UNICEF: United Nations International Children’s Emergency Fund
VAPP: Violence Against Prohibited Persons
